# Role of Seed Layer Crystalline Quality in Photoresponse Performance of Hydrothermally Grown ZnO Nanorods

**DOI:** 10.3390/ijms23179541

**Published:** 2022-08-23

**Authors:** Waqar Muhammad, Kiyun Nam, Songji Seo, Sam-Dong Kim

**Affiliations:** Division of Electronics and Electrical Engineering, Dongguk University, Seoul 100-715, Korea

**Keywords:** ZnO, seed layer, nanorod, hydrothermal growth, p-n photodiode, surface defects

## Abstract

We investigated the effects of the crystalline state for seed layers (SLs) on the growth morphology and material characteristics for hydrothermally grown ZnO nanorods (NRs). For this, preheating (PH) at different temperatures (100–300 °C) and O_2_ plasma treatment (PT) for 9 min were performed during the growth of SLs on p-Si by the aqueous solution-based method to provide the characteristic change on the NR growth platform. An improvement in material properties was achieved from the ZnO NRs grown on the SL crystals of enhanced crystalline quality in terms of the increased preferred orientation (002), the higher UV emission with suppressed deep-level emissions, the recovery of O/Zn stoichiometry, and the reduction of various intrinsic defects. Ultraviolet photodiodes of a p-Si/n-ZnO-NR structure fabricated under the SL conditions of O_2_ PT and PH at 100 °C showed a significantly enhanced on-off current ratio of ~90 at +5 V and faster photoresponse characteristics presenting a reduction in the fall time from 16 to 9 s.

## 1. Introduction

A great amount of research efforts has been made to investigate ZnO nanostructured materials in recent decades due to their substantially attractive performance in electronics, optics, and photonics. Having a high surface-to-volume ratio, they introduced the new challenges in electrical, mechanical, chemical, and optical properties with quantum confinement effects; therefore, these unique morphological structures of ZnO have found their novel application areas in sensors, photodetectors, transducers, and photo-catalysts [1,2]. The bottom-up approach of the ZnO nanostructure synthesis method has been investigated in a variety of ways, such as thermal decomposition, sputtering, vapor phase epitaxy, molecular beam epitaxy, co-precipitation, and pulsed laser deposition [3,4,5,6]. Although these methods have explored and provided state-of-the-art technologies suitable for their future applications of ZnO nanostructures, but most of them are limited in many practical aspects, such as high temperature growth which is not allowed for organic substrates, textile fibers or plastic polymers, and vacuum-based sophisticated laboratory facilities which are not proper for the large-area growth. The hydrothermal process recently has gained immense interest in growing ZnO nanorods (NRs) due to its simplicity and tolerable growth conditions, and this process is based on the synthesis method in aqueous solutions which has been extensively examined in many ways [7]. Moreover, we have numerous advantages in the solution-based method in terms of easy incorporation of dopants, process reproducibility at low temperatures, and desirable homogeneity in large-size growth, without using sophisticated equipment. However, we have to overcome some critical issues such as the incorporation of impurities and native defects to assure high-quality hydrothermal ZnO crystals grown by this method suitable for the device applications.

A variety of factors can influence on the crystallinity of hydrothermally grown ZnO nanostructures such as substrate materials and their preparation, solution chemistry, heat treatment condition, and growth time. It was revealed by earlier experimental report [8] that the preparation conditions for the ZnO seed layer (SL) can play a critical role in the consequent material quality of ZnO NR arrays grown by the vapor phase transport technique. Our research efforts were made in this study to explore effective synthesis and perform investigations on how the growth mode and material quality of hydrothermal NRs could be controlled by the quality of SL crystallization. For this purpose, extensive material characterizations were performed on ZnO SLs synthesized by multi-step spin coatings at various preheating (PH) temperatures in a range of 100–300 °C and oxygen plasma (O_2_) treatment (PT) producing many high-energy radicals of high diffusivity in ZnO. The coated SL films formed in the amorphous state by the sol-gel process were converted into the crystalline state during the PH step and by the final post-annealing process in general. We explored the effects of the SLs PH temperature on the material property of ZnO NR arrays grown by the hydrothermal method on the SLs. Because the sol-gel ZnO NR growth on flexible substrates needs to proceed at relatively low temperature below ~300 °C, understanding the effects of PH conditions on the crystalline characteristics of SLs and NRs at this low temperature range has a great significance. We fabricated ultraviolet (UV) photodiodes with a p-Si/n-ZnO-NR heterojunction structure and examined the key device parameters influenced by the NR material properties to confirm our material characterization on the improvement of NR crystalline quality by the SL treatment.

## 2. Results and Discussion

**Figure 1:**
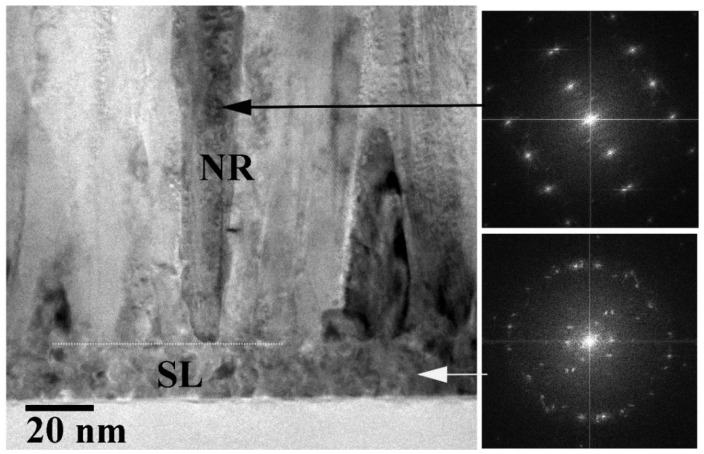
Cross-sectional high-resolution TEM image of the ZnO NRs grown on an SL (PH at 100 °C, no PT). Electron diffraction patterns are also shown from the selected areas of NRs (**top-right**) and the SL (**bottom-right**).

**Figure 2:**
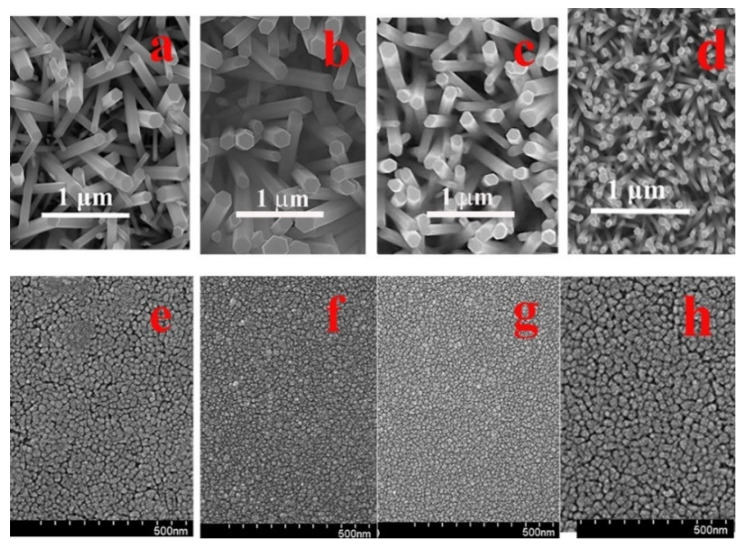
Cross-sectional SEM views of the NRs grown on the as-coated SLs preheated at 100 °C (**a**), 200 °C (**b**), and 300 °C (**c**) and O_2_-plasma-treated SLs (PH at 100 °C) (**d**). Plane-view SEM micrographs of the ZnO SLs preheated at 100 °C (**e**), 200 °C (**f**), and 300 °C (**g**) and O_2_-plasma-treated SLs (PH at 100 °C) (**h**).

The cross-sectional cross-section transmission electron microscopy (TEM) analysis on the control sample (CS, PH at 100 °C and no O_2_ PT for SL) revealed the growth of nano-crystalline SLs (~20 nm) comprising very fine grains with a few tens of nanometers in diameter, as shown in Figure 1. The ring-shaped diffraction pattern from the selected area of SL (the bottom right of Figure 1) also exhibited the polycrystalline structure of a fairly random orientation. On the other hand, we observed a clear [11$\bar{2}$0] zone-axis diffraction pattern of hexagonal ZnO single crystals from the NRs (the top right of Figure 1). The grown NR crystals were structurally uniform and aligned to the [2] direction (c-axis) normal to the substrate. As observed from the scanning electron microscopy (SEM) micrographs in Figure 2a, the CS exhibited widespread diameters of the NRs (80–220 nm) with a mean diameter of ~160 nm and an average length of ~1.7 μm. We summarized in Table 1 the dimensional characteristics of the NRs measured by SEM analysis, and 50 NRs in total were taken into account from four different sample positions for this measurement. The aspect ratio and the surface density of the NRs were ~10 and ~25 rods/μm^2^, respectively, in the case of CS. From the NRs grown on the plasma-treated SL, a great amount of changes in NR morphology, such as the diameter, length, aspect ratio, and rod density, were obtained as shown in Figure 2d and Table 1. We observed a much smaller mean diameter of ~65 nm and a more uniform diameter distribution of 50–80 nm (the reduction of standard deviation from 7.2 to 5.2). Due to the enhanced (002) growth mode, a much higher aspect ratio of ~28 and a much higher surface density of ~80 rods/μm^2^ were also obtained. This accelerated NR nucleation rate and the enhancement in longitudinal growth mode induced by O_2_ PT can be attributed to the oxidation of Zn interstitial atoms caused by the PT process as proposed by Hou et al. [9].

The influences of the PH temperature on the NR morphology are also shown in Table 1. A reduced aspect ratio and a decreased NR density were obtained with the increase of the PH temperature for SLs, and this phenomenon can be associated with the change in the crystalline state of SLs, as shown in Figure 2e–h. The SL grain size was reduced from 29 to 21 and 19 nm with the increase of the SL PH temperature from 100 to 200 and 300 °C as measured with the planimetric method by measuring the number of grains per unit area [10]. This is an opposite trend with the common observation of a larger grain size at a higher thermal budget due to the promoted grain growth caused by more thermal energy available for the atomic inter-diffusion and the coalescence between the neighboring grains. In this case, our PH thermal budget (100–300 °C, 1 min) was not so high enough to promote active grain growth and intergranular coalescence through thermal diffusion. More importantly, the instant vaporization of almost all components in the SL sol (including zinc acetate) could be made with the increase of the PH temperature, thereby disturbing the proper ZnO nucleation and relaxation along the preferred orientation of (002) and minimizing the growth time for the crystal nuclei.

**Figure 3:**
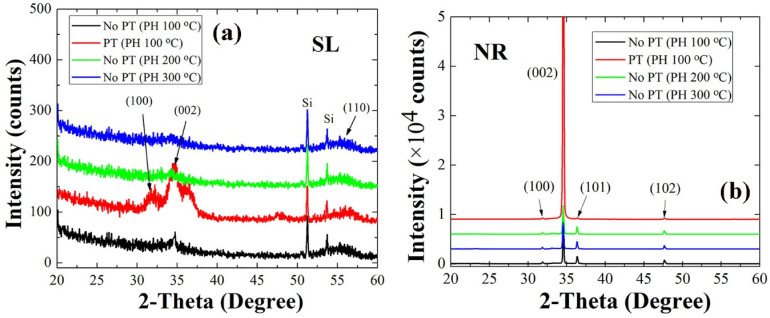
XRD patterns (2θ scan) of ZnO SLs (**a**) and NRs grown on the SLs (**b**) prepared under different conditions.

The relationship of the SL crystalline state with the growth mode of NR crystallites observed by SEM were examined in more detail through our X-ray diffraction (XRD) analysis. Shown in the XRD spectra of Figure 3 are all the principal reflections from the SLs and NRs, and they showed a good agreement with the indexes of JCPDS files for the hexagonal phase ZnO (card number: 36-1451). As summarized in Table 2, the dominant (002) peak intensity recorded from the NR crystals were decreased with the increase of the PH temperature; however in particular, a remarkable increase by ~11 times was obtained in the case of O_2_ PT. The degree of the (002) orientation also followed the same dependence on the SL condition and exhibited a very high degree of the (002) orientation **F**(002) (~0.95) in the case of PT. One notes that this is due to the enhancement in the preferred (002) orientation of the SL nano-crystallites as revealed in XRD patterns of Figure 3a. The plasma-treated SLs showed an intense peak of (002) at 2θ = 34.5° with very weak reflections from other planes such as (100) and (110). On the other hand, much reduced (002) intensities were recorded from all the untreated SLs preheated at different temperatures. The (002) growth mode of ZnO NRs has the lowest surface free energy (~1.6 J/m^2^) among other crystal plane growth modes such as (100) and (102) (~3.4 and ~2.0 J/m^2^, respectively) [11]; therefore, this growth mode can be promoted with the lowest activation energy when grown on the nanocrystalline SLs of a more highly preferred orientation toward (002). **F**(002) of NR crystals was also slightly reduced with the increase of the PH temperature, which is also related to the effect of the SL grain size and the preferred orientation made by different PH conditions. The enhancement of vertical NR growth modulated by the SL crystalline state can be also associated with the suppressed surface contamination and structural defects [12,13], and the further details of this phenomenon were discussed in our X-ray photoelectron spectroscopy (XPS) analysis.

**Figure 4:**
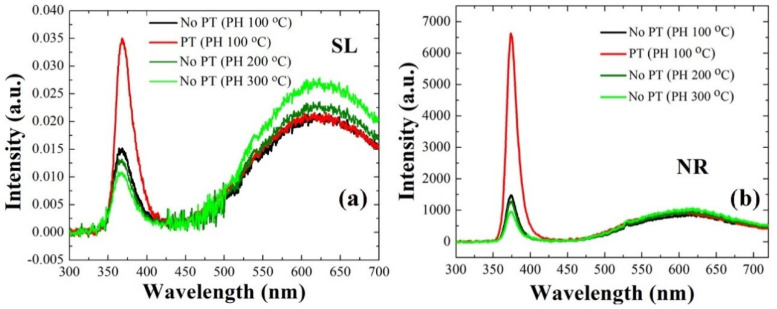
Room-temperature PL spectra of ZnO SLs (**a**) and NRs grown on the SLs (**b**) prepared under different conditions.

Photoluminescence (PL) analysis was performed on the ZnO NRs grown with the SLs prepared under different conditions, and the optical properties of the nano-crystallites were examined as depicted in Figure 4. Among various emission patterns, the UV emission centered at ~374 nm in wavelength is attributed to the band-to-band excitonic recombination which depends on the density of defects causing the intraband transitions. Broadband visible emission (450–600 nm, green-yellow radiation), on the other hand, are known to be linked to the deep-level emissions (DLEs) and the recombination of photo-generated carriers with various types of intrinsic defects in ZnO [14,15,16,17]. As shown in Figure 4b, a ~4.6 times higher UV emission was obtained from the NRs grown on plasma-treated SLs than the emission from the CS, which is attributed to the improved crystalline properties (see Figure 4a) of the SL materials serving as a growth platform for the NRs. With the increase of the PH temperature from 100 to 200 and 300 °C, the UV emission was reduced to ~87% and ~65%, respectively, of the emission from the CS. On the contrary, broad green-yellow emissions from the SLs were significantly suppressed with the PT or in the case of PH at 100 °C (no PT). The exact location for DLEs associated with various intrinsic defects and their exact nature have not been fully understood and still in debate [18]. However, these broad emissions are known to be caused by various non-stoichiometric intrinsic defects in the chemically grown ZnO [19], and they can be also originated from the zinc vacancy and/or anti-site defects [20].

**Figure 5:**
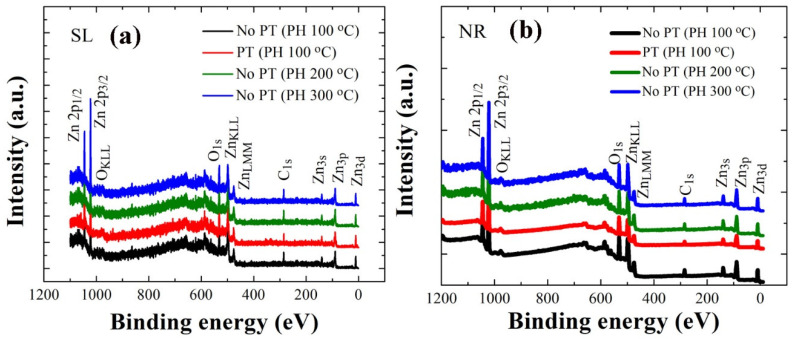
XPS wide scan spectra of ZnO SLs (**a**) and NRs grown on the SLs (**b**) prepared under different conditions.

**Figure 6:**
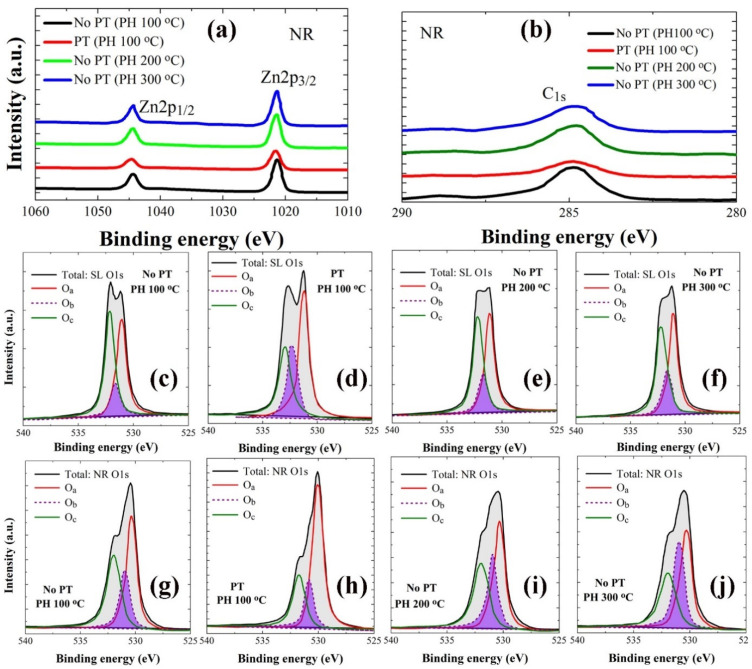
High-resolution individual core-level spectra of Zn 2p (**a**) and C 1s (**b**) from the NRs grown on the SL prepared under different conditions. Gaussian deconvoluted O 1s spectra from the SLs preheated at 100 °C (**c**), 200 °C (**d**), and 300 °C (**e**) and O_2_-plasma-treated SLs (PH at 100 °C) (**f**). The deconvoluted O 1s spectra from the NRs grown on the SLs prepared at each condition of (**c**−**f**) are also shown in (**g**–**j**).

XPS analysis was performed in a binding energy (BE) range of 0–1200 eV to examine the stoichiometry and chemical state of the constituent elements in the ZnO crystals as, shown in wide scan spectra of Figure 5. From each layer of seeds and NRs, a variety of photoelectron peaks with spin-orbital splittings were recorded with additional Auger peaks. The peaks from Zn element in the ZnO NRs are shown in Figure 6a with two different core-level Zn peaks (2p_1/2_ at 1044.3 eV and 2p_3/2_ at 1021.2 eV) separated by spin-orbital splitting. In addition to the peaks from main constituents of Zn and O, some traces of carbon were also detected. C1s spectra in Figure 6b are in general caused by the surface contamination arising from hydrocarbon and carbon oxides during the solution-based synthesis process. Shown in Figure 6c–f and 6g–j are the asymmetric high-resolution O1s peaks of the SL and NR samples prepared under different conditions, respectively. Gaussian peak deconvolutions for each O1s curve can give three distinctive satellite peaks at 530.0 eV (Oa), 531.0 eV (Ob), and 531.8 eV (Oc), as shown in Figure 6c–j. Oa is known to be caused by O^2−^ ions in the hexagonal Zn^2+^ ion array surrounded by the wurtzite Zn crystal matrix [21,22]; therefore, the contribution of this peak can be a good measure for the amount of oxygen atoms in a fully oxidized stoichiometric surrounding. The Oc spectrum at a higher BE of 531.8 eV is attributed to chemisorbed or dissociated oxygen or OH species on the surface of the ZnO thin film, such as −CO_3_, adsorbed H_2_O, or adsorbed O_2_ [22,23]. The Ob spectrum at a BE of 531.0 eV can be associated with O^2−^ ions in the oxygen-deficient regions within the matrix of the ZnO film [24]; therefore any change in this component may be connected in part to the variations in the concentration of oxygen vacancies [22]. Since Oa comes from the stoichiometric oxygen in ZnO, we can evaluate the O/Zn stoichiometry by calculating ∫Oa/∫Zn, where ∫Oa and ∫Zn represent the peak curve integrations of Oa and Zn in XPS spectra, respectively. The stoichiometric oxygen contribution to total oxygen in ZnO can be evaluated by ∫Oa/∫Ot, where ∫Oa and ∫Ot represent the peak curve integrations of Oa and total O1s, respectively. Similarly, the contributions of Ob and Oc were extracted by ∫Ob/∫Ot and ∫Oc/∫Ot, respectively, in each case. Atomic percentages of carbon were also estimated using the quantitative analysis by integrating the total area under the C1s curve. All these extracted parameters are summarized in Table 3.

The carbon percentage (at.%) of the SL was reduced from 21.3% (no PT) to 19.4–20.4% with the increase of the PH temperature; however, in the case of PT, it was significantly dropped to 16.3%. Residual carbon in the SLs was projected in the same trend to the carbon amount in the subsequently grown NR crystals as shown in Table 3. The as-grown ZnO normally exhibited oxygen deficiency in nature caused by a high density of oxygen vacancies; however, the O_a_/Zn ratio of the NR crystals was increased from 0.61 (CS) to 0.91, when the SLs were plasma-treated due to the effective enhancement in SL stoichiometry caused by the oxygen supply from the plasma state. The percentage of Ob in the SL was also increased from 11.2% to 21.1%, but the Oc percentage (%) was greatly suppressed from 51.7% to 17.3% most probably by the effective elimination of undesired oxygen-bonded species such as adsorbed water, chemisorbed O_2_, and other oxygenated impurities present at the surface or grain boundaries of the SL crystals. Based on this result, more active intergranular coalescence can be expected with grain growth via an accelerated diffusion process along grain boundaries, which give rise to a more stable (002) preferential orientation in the SL crystals in the post-annealing process. The growth process with the lowest activation energy can be thus achieved, when the NR crystals grow on the high crystalline-quality SLs of minimized defects and well-aligned grain orientation and tend to preferentially align perpendicular to the substrate surface.

**Figure 7:**
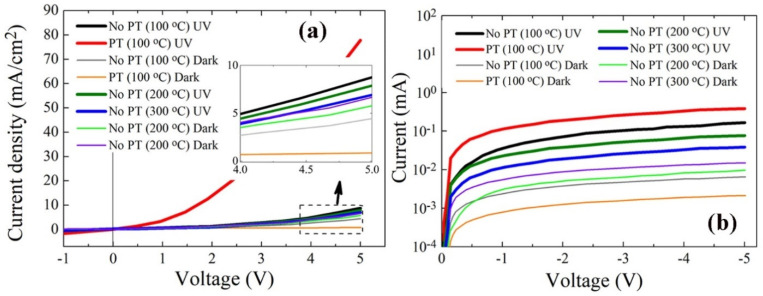
Typical diode photoresponse I−V characteristics of p-Si/n-ZnO NRs grown on the SLs prepared under different conditions according to UV illumination on/off in a forward bias range of −1 ± 5 V (**a**) and a reverse bias range of −5–0 V (**b**).

The crystal quality in the active region of photodiode delivers a significant impact on the device performance. In particular, the desorption kinetics of oxygen ions on the surface of ZnO NRs active region is directly related to the photo-carrier generation under UV illumination, and various intrinsic and extrinsic types of defects in ZnO nano-crystallites are also deeply involved in this process. Figure 7 shows the I−V characteristics for the typical rectifying behavior of the p-n photodiode in a voltage range from −5 to +5 V, and key parameters extracted from the measurements are listed in Table 4. At a forward bias of +5 V, a great amount of increase in the on-off current ratio I_on_/I_off_ (~90.5) was obtained from the photodiodes fabricated with the ZnO NRs grown on the plasma-treated SLs, whereas a much lower I_on_/I_off_ of ~2.0 was measured from the CS diodes (no PT and PH at 100 °C for SLs). Even under a reverse bias of −5 V, the I_on_/I_off_ from the diodes with the NRs grown on plasma-treated SLs was 7.6 times greater than that of the CS diodes. The effect of the PH temperature on the device performance also followed the same direction with our material characterization of ZnO NRs, and the highest of I_on_/I_off_ values at both +5 V and −5 V were obtained at a PH temperature of 100 °C.

A huge increase of the photocurrent in diodes by the PT is the main contributor to the enhanced I_on_/I_off_ in a forward bias condition. The origin of this photocurrent enhancement can be attributed to the modulation of the channel conductivity through the ZnO NRs under UV illumination. In the dark state, oxygen molecules are chemisorbed to the ZnO surface by capturing electrons as shown in the reaction of Equation (1), and a low-conductivity depletion layer is formed near the surface of ZnO which was originally n-type by the presence of many oxygen vacancies and Zn interstitials [12,25]. When UV illumination commences, the desorption process for the O_2_^−^ ions starts to proceed, as shown in Equation (2), with the electron−hole pair generation in the near surface of ZnO. Consequently, the surface depletion region near the NR surface starts to shrink, so that the NR channel conductivity is further enhanced. However, deep-level defects present on the ZnO surface tend to trap the photogenerated carriers and suppress this modulation of surface depletion. The increase in I_on_/I_off_ by the SL PT under forward bias conditions can be explained by the healing effect of deep-level defects on the NR surface and the consequent lowering of surface band bending.

(1) $$O_{2}+{\;e}^{-}\;\to {\;O}_{2}^{-}\;\;\to (\mathrm{dark}),$$

(2) $$O_{2}^{-}+{\;h}^{+}\;\to {\;O}_{2}^{\;}\;\;\to (\mathrm{under}\;\mathrm{UV}).$$

The enhancement of I_on_/I_off_ under reverse bias conditions is due to both the reductions of I_off_ by approximately three times and the increase of I_on_ by ~two times from the diodes prepared with SL PT than that of CS, as shown in Table 4. The reduction of I_off_ was not clearly understood, but it can be associated with the experimental observation of the increase in the ZnO sheet resistance after O_2_ PT [26]. This gives rise to the reduction in the free carrier concentration and the electrical conductivity in the current path between p-Si and n-ZnO NRs by the diffusion of oxygen radicals through the ZnO SLs and compensation of oxygen vacancies.

**Figure 8:**
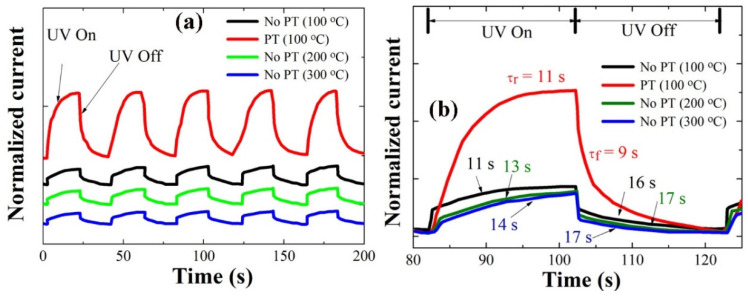
(**a**) Transient characteristics of p-Si/n-ZnO-NRs heterojunction PDs grown on the SLs prepared under different conditions; (**b**) the expanded views of the photoresponses in a single cycle in (**a**).

We measured the transient responses of the photodiodes at a forward bias of +5 V with a time lapse of 20 s upon the real-time switching of UV light illumination and turn-off using a light source of 254 nm wavelength and a 7 mW/cm^2^ light intensity. As shown in Figure 8a, all the diodes fabricated under different SL conditions showed a fairly good repeatability. The diode fabricated with plasma-treated SLs showed almost the same rise time **τ_r_** (~11 s), but a much shorter fall time **τ_f_** (~9 s) than that with the CS (~16 s) despite a much larger photocurrent upon the UV illumination (see Figure 8b), where the rise time and the fall time (or decay time) were given by time intervals for the photocurrent to rise up to 90% of the maximum saturation value after UV turned on and for the photocurrent to fall off by 90% from the maximum value after UV illumination was turned off. The response time according to the SL PH temperature did not show a significant change but followed the same trend with our material characterization for the NR crystalline quality, and the fastest response speed was obtained in the case of PH at 100 °C. The transient response of our photodiodes can be influenced by many factors in the serial process for the oxygen adsorption and desorption on the ZnO surface, but the impact induced by the change in the NR crystal quality can be associated with the surface band bending near the surface depletion region. When the surface band bending is lowered by the effective suppression of deep-level defects, the electron diffusional flux toward the NR surface region to produce adsorbed O_2_ ions is exponentially increased with the reduction of the electron energy barrier. Because the recovery process is under control by the electron diffusion across the surface bending and the consequent oxygen adsorption, the improvement of the recovery speed can be expected from the SL PT technique.

## 3. Materials and Methods

### 3.1. Fabrication of Materials and Devices

**Figure 9:**
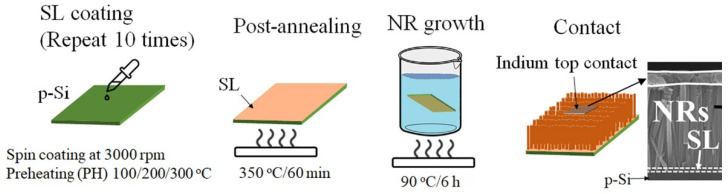
Schematic illustration of the process flow for the UV photodiode with a heterojunction structure of p-Si/n-ZnO NRs grown on as-coated (PH at 100, 200, and 300 °C) and O_2_-plasma-treated (PH at 100 °C) SLs.

p-n heterojunction photodiode structures were fabricated by growing n-ZnO NRs on boron-doped p+ Si (100) substrates (1.2 × 1.2 cm^2^, 0.01 Ω cm resistivity; 4Science, Seoul, Korea), as depicted in the process flow of Figure 9. All the chemicals used in this work were of a reagent grade purchased from Sigma-Aldrich, Seoul, South Korea. After cleaning the Si substrates by acetone, isopropyl alcohol, and de-ionized (DI) water in sequence, the SL growths were performed in the following way. A sol-gel solution for the SL growth was prepared by mixing zinc acetate dehydrate [Zn(CH_3_COO)_2_∙2H_2_O] with 1-propanol to obtain a concentrate molarity of 20 mM. After sonicating the solution for 30 min to achieve agglomerate-free sol-gel mixing, the SL solution was spun onto the Si substrates at 3000 rpm for 30 s in a spin coater. This spin coating process was repeated 10 times to attain a final SL thickness of ~20 nm as revealed by the TEM image in Figure 1. After each single coating step, PH for the SLs was performed at three different temperatures of 100, 200, and 300 °C for 1 min to investigate the effects on the NR crystalline quality. The SLs were then post-annealed at 350 °C for 10 min, and the remaining unwanted organic compounds and water in the grown SLs were removed as much as possible in this step. To examine the effects of O_2_ plasma exposure to the SL crystallites, PT was performed for the SLs of CS (PH at 100 °C) under a O_2_ flow rate of 100 sccm, an RF plasma power of 100 W, and a process pressure of 50 mTorr in a reactive ion etcher for 9 min. After completing the SL growths under different conditions, the ZnO nano-crystallites were grown by the hydrothermal method in a solution of 25 mM equimolar zinc nitrate hexahydrate [Zn(NO_3_)_2_·6H_2_O] and hexamethylenetramine [C_6_H_12_N_4_]. For this, the substrates were dipped in the growth solution (fully agitated using an ultrasonic for 5 min) and maintained at 90 °C for 7 h. The samples were finally rinsed with DI water, purged with nitrogen gas and experienced a subsequent final bake at 120 °C for 2 min.

### 3.2. Characterizations

The dimensional characteristics and the surface morphology of the as-grown ZnO NRs and SLs were characterized by SEM (Hitachi S-4800S at 15 kV) and TEM (9500-Hitachi with 500 KV). The XRD 2θ patterns of the SL and NR samples were recorded using a D8 Advance spectrometer of Bruker AXS with Cu K_α_ radiation (λ = 0.1540 nm). From this XRD analysis, we calculated the degree of (002) orientations **F**(002) for the grown NR crystals by the following relationship [27]:

(3) $$F\left(002\right)=\left[P\left(002\right)-P_{o}\left(002\right)\right]/\left[1-P_{o}\left(002\right)\right],\;$$

where P(002) = I(002)/ΣI(hkl) and *P_o_*(002) = I_o_(002)/Σ(I_o_(hkl), where I(002) is the measured peak intensity from the (002) plane and I_o_(002) is the reference peak intensity of the same index plane given by JCPDS card No. 36-1451. We performed XPS for the analysis of the chemical bonding states and the stoichiometric information of the nano-crystallites using a PHI 5000 Versa Probe (Ulvac-PHI) spectrometer and a monochromator Al K_α_ (1486.6 eV) anode (25.0 W, 15 kV). Optical properties of the crystals were examined by room-temperature PL spectroscopy (MFP-3D Bio, Asylum Research) excited at 266 nm.

The photoresponse I−V characteristics of the photodiodes were measured using a Keithley 2400 source meter unit (Beaverton, OR, USA) and a short-arc Xe lamp light source operating at 300 W according to the UV illumination. The light of a wideband (200–800 nm) produced by a Xe lamp (model 300XF-R1) was guided to the filter unit with a monochromator (CM11 1/4 m) and a grating of 2400 lines per millimeter, and a filtered light of 254 nm wavelength was used for the input light in the photoresponse measurement. For the transient measurements, the input UV light was controlled by a programmable electronic shutter, and all the measurements were carried out in a black box to prevent any interference of ambient light. As illustrated in Figure 9, the I–V characteristics of the diodes were measured across an indium metal contact (~1 mm^2^) pasted on the NRs and a direct contact to p-Si. A good specific contact resistance of ~1 × 10^−2^ Ω cm^2^ was measured from our indium pasting with no additional alloy.

## 4. Conclusions

In this study, the effects of the SL crystalline quality on the structural, optical, and electrical properties of hydrothermally grown ZnO NRs were investigated according to the PH temperature and O_2_ plasma irradiation for the SL crystallites grown by the aqueous solution-based method. The increase of the SL PH temperature resulted in the deterioration of the SL crystalline quality in all aspects, such as (002) directional growth, optical properties, O/Zn stoichiometry, and residual carbon. On the other hand, the NR crystals grown on the SLs prepared through O_2_ PT showed a significant improvement in physical, optical, and chemical properties. It was found that the PT process restored the stoichiometry of ZnO SL crystals by minimizing the various crystalline defects such as oxygen deficiency, oxygen-containing impurities, and oxygenated carbons arising from the growth solution. The ZnO NRs grown on the SLs of the enhanced crystalline quality showed a remarkable improvement in material characteristics in terms of the increased degree of (002) growth orientation, the higher UV emission with the reduced emission from the deep level states, the restoration of O/Zn stoichiometry, and the suppression of various intrinsic defects. The performance p-Si/n-ZnO-NR UV photodiodes followed the same direction with the SL crystalline quality, and a great increase of I_on_/I_off_ and a faster response speed especially in the recovery process (upon UV turn-off) were measured from the devices fabricated with the NRs grown on the plasma-treated SLs.

The improvement in the device performance is due to the enhanced crystalline properties of ZnO nano-crystallites and the effective suppression of various deep-level defects, which are the key to the performance of photonic device. The synthesis method and the analysis presented in this article can be further extended to other metal oxide nanostructures synthesis on flexible substrates with low-cost and large-scale production.

## Figures and Tables

**Table 1:** Summary of the morphology statistics measured from the SEM images of ZnO NRs grown on the as-coated (PH at 100, 200, and 300 °C) and O_2_-plasma-treated (PH at 100 °C) SLs.

PT and PH Conditions for SLs	Mean Diameter (nm)	Average Length (μm)	NR Density (rods/μm^2^)	Aspect Ratio
No PT (PH at 100 °C)	160	1.67	25	~10.4
No PT (PH at 200 °C)	171	1.58	22	~9.2
No PT (PH at 300 °C)	174	1.49	21	~8.6
O_2_ PT (PH at 100 °C)	65	1.84	80	~28.3

**Table 2:** Parameters of ZnO NR crystals extracted from XRD analysis.

PT and PH Conditions for SL	2θ of (002) (Deg.)	(002) Intensity (Normalized)	FWHM (Deg.)	F(002)
No PT (PH at 100 °C)	34.56	1	0.19	0.67
No PT (PH at 200 °C)	34.54	0.87	0.20	0.64
No PT (PH at 300 °C)	34.54	0.84	0.20	0.61
O_2_ PT (PH at 100 °C)	34.56	11.2	0.17	0.95

**Table 3:** Percentage values of the XPS O1s satellite peaks from the as-coated (PH at 100, 200, and 300 °C) and O_2_-plasma-treated (PH at 100 °C) SLs and NRs grown on each SL crystal. Each percentage value was calculated by dividing the curve integration of individual satellite peak by the curve integration of the total peak.

PT and PH Conditions for SL		Oa (%)	Ob (%)	Oc (%)	Oa/Zn	C (at.%)
SL	No PT (PH at 100 °C)	37.1	11.2	51.7	0.61	21.3
	No PT (PH at 200 °C)	36.4	14.5	49.1	0.57	20.4
	No PT (PH at 300 °C PH)	35.6	19.1	45.3	0.53	19.4
	O_2_ PT (PH at 100 °C)	61.5	21.1	17.3	0.86	16.3
NR	No PT (PH at 100 °C)	57.4	14.8	27.8	0.79	6.8
	No PT (PH at 200 °C)	54.2	19.5	26.3	0.73	6.3
	No PT (PH at 300 °C)	52.1	23.8	24.1	0.65	5.9
	O_2_ PT (PH at 100 °C)	61.9	12.7	21.2	0.91	3.4

**Table 4:** Summary photocurrents, dark currents, and on-off current ratios at bias conditions of +5 and −5 V measured from the p-Si/n-ZnO-NR heterojunction PDs.

Parameter	PT and PH Conditions for SLs	Forward Bias (+5 V)	Reverse Bias (−5 V)
I_on_ (mA/cm^2^)	No PT (PH at 100 °C)	8.76	1.80
	No PT (PH at 200 °C)	7.89	0.83
	No PT (PH at 300 °C)	6.92	0.41
	O_2_ PT (PH at 100 °C)	77.86	4.11
I_off_ (mA/cm^2^)	No PT (PH at 100 °C)	4.45	0.070
	No PT (PH at 200 °C)	5.79	0.082
	No PT (PH at 300 °C)	6.68	0.094
	O_2_ PT (PH at 100 °C)	0.86	0.021
I_on_/I_off_	No PT (PH at 100 °C)	1.97	25.71
	No PT (PH at 200 °C)	1.34	10.12
	No PT (PH at 300 °C)	1.04	4.36
	O_2_ PT (PH at 100 °C)	90.53	195.71
